# Longitudinal and Other Sinus Thrombosis

**Published:** 1908-01-11

**Authors:** 


### Illustrative Cases.

### LONGITUDINAL AND OTHER SINUS THROMBOSIS.

Sinus thrombosis within the cranium is nearly always secondary to one or other of two groups of causes, namely:—

(1) Infective inflammation in the neighbourhood of the sinus, which becomes thrombosed; for example, lateral sinus thrombosis secondary to otitis media.

(2) Cachexia, whether due to simple marasmus, or to such causes as phthisis, Bright's disease, malignant disease, tropical and other fevers, syphilis, or a blood disease.

When the sinus thrombosis occurs as the result of any of the above, not only is one alive to the fact that it may occur, but also the symptoms due to it are mingled with the symptoms of the other troubles that are present. It does not often happen that one meets with an adult in whom the sole lesion is thrombosis of longitudinal, lateral, and occipital sinuses. The following case may be of interest, therefore, as indicating the symptoms due to sinus thrombosis itself. It is noteworthy how closely they simulate those of intracranial tumour.

### An Illustrative Case.

A full account of the condition is given by Dr. C. Eugene Riggs in the *Journal of Nervous and Mental Diseases*. The patient was an unmarried lady, aged twenty-two, whose occupation was that of a school-teacher. She had been perfectly well until one day she was thrown out of a conveyance in mid-winter, striking her head badly upon the hard ground. This, naturally, gave her a severe headache for a few days, and she vomited shortly after the accident; but she soon recovered, and she seemed to remain in thoroughly good health until eighteen months later. She then began to suffer from severe headaches, which were regarded as neuralgic. The pain was worst in the right frontal region, and simultaneously with it the face was pale, the eyelids were slightly œdematous, and the eyes were surrounded by dark rings.

The headaches were at first periodic, though they were never absent for longer than three days at a time. Later she was never free from them, and there were exacerbations, which were nearly always in the daytime and mostly in the afternoon. About three weeks after the beginning of this illness she had such violent pains in the head that she had to stay in bed, where she remained continuously for a fortnight; and for the first two days of this fortnight she was repeatedly sick. Later she was up and about, but quite incapacitated from work, and whenever the exacerbations in the headache occurred she vomited.

Six months later she had almost lost her eyesight. There was well-marked optic neuritis in both eyes, of the choked-disc variety; she could not distinguish the number of fingers one held up before her. Intelligence was unimpaired. There had been no convulsions. Her temperature was normal. The pulse-rate varied a little above and a little below 70 per minute. The urine was natural. The pupils were dilated; they reacted to light and to accommodation. There was no inco-ordination, no sensory and no vasomotor disturbance. The plantar reflexes were both flexor. The only abnormality in the peripheral nervous system was that the right knee jerk was more pronounced than the left.

A month later there was some twitching in the right side of the neck, confined to the sterno-cleido-mastoid and platysma myoides muscles. Later a similar twitching was noticed on the left side now and then, and it was observed sometimes to spread to the muscles of both sides of the face. This was the nearest approach to anything like a convulsion.

It was not surprising that cerebral tumour was diagnosed. There were no localising symptoms, for the twitchings of the face and neck were bilateral, and there was no paralysis. Nevertheless, it was clear that the only way to give the patient any relief at all was to trephine in order to decrease the pressure upon the brain. There was not the slightest evidence of syphilis; mercury and potassium iodide could do nothing.

The skull was therefore trephined, and for several weeks afterwards the pains in the head absolutely disappeared. Nothing was seen, even at the operation, to suggest that the lesion was other than a tumour. Relief was temporary only, however. After a few brief weeks the pains in the head recurred with fiendish intensity. A second attempt to alleviate them by trephining was made, but the patient died during the attempt.

### Post-Mortem Findings.

At the post-mortem examination there was no intracranial growth. The brain and the cerebellum, their peduncles, the pons varolii and the medulla oblongata were all perfectly healthy-looking. The longitudinal and two lateral sinuses were almost completely obliterated. The adhesions between the sinus walls were old and inseparable. Here and there in the course of the longitudinal sinus there was an open space corresponding to the entrance of a vein. The torcular Herophili and the occipital sinuses were obliterated in the same way. There were some shreds of a brownish colour in some of the unobliterated foci in the superior longitudinal sinus; these seemed to be blood-clots. In the parts of the sinuses which were obliterated entirely the clot was fibrosed. No cause for the thrombosis was found. It may possibly be attributed to the head injury, a small clot at the time being followed later by an extension of the process. There is, however, no proof of this.

The interest of the case lies mainly in the fact that the thrombosis gave all the symptoms of a cerebral tumour, namely, headache, vertigo, vomiting, optic neuritis, twitchings; and we especially lay stress upon the optic neuritis occurring as the result of a purely vascular condition.

